# Histopathological patterns of thyroid lesions in a teaching health facility: an 11-year review

**DOI:** 10.4314/ahs.v26i1.13

**Published:** 2026-03

**Authors:** Adebayo Ayoade Adekunle, Olabisi Ayo-Aderibigbe, Najeem Adedamola Idowu, Mumini Wemimo Rasheed, Afolabi AbdulKareem Salawu, Saliu Adetunji Oguntola

**Affiliations:** 1 Department of Morbid Anatomy and Histopathology, Ladoke Akintola University of Technology, Ogbomoso, Nigeria; 2 Department of Surgery, Ladoke Akintola University of Technology, Ogbomoso, Nigeria; 3 Department of Anatomic Pathology, Federal University, Dutse, Jigawa State, Nigeria; 4 Department of Chemical Pathology, Ladoke Akintola University of Technology, Ogbomoso, Nigeria

**Keywords:** lesions, thyroid, carcinoma, goitre, histopathology, patterns

## Abstract

**Background:**

Thyroid diseases vary geographically, with iodine deficiency being a major cause in Africa. These conditions range from congenital anomalies to acquired neoplastic and non-neoplastic lesions.

**Aim:**

To establish baseline data on the frequency and patterns of thyroid lesions diagnosed in a teaching hospital's histopathology department over an 11-year period (2012–2022).

**Methods:**

A retrospective descriptive study was conducted using slides and paraffin-embedded blocks of thyroidectomy specimens from January 2012 to December 2022. Clinical data including age, sex, and histological diagnosis were retrieved and analyzed using SPSS version 23.0.

**Results:**

A total of 111 thyroid lesions (2.4% of all cases) were reviewed, with a female-to-male ratio of 5.5:1 and a mean age of 42.5 years (SD = 12.94). Hyperplastic lesions predominated (76.6%), comprising nodular hyperplasia (45.0%) and diffuse goitre (31.5%). Neoplasms accounted for 18.9%, including papillary carcinoma (7.2%) and follicular carcinoma (4.5%). Congenital and inflammatory lesions were least frequent. Neoplasms peaked in the fourth decade, with significant associations between age, gender, and lesion type (p < 0.001).

**Conclusion:**

Thyroid lesions showed strong female predominance. Non-neoplastic conditions were most common, with peak incidence between the fourth and sixth decades.

## Introduction

The thyroid gland is an endocrine organ that controls various metabolic processes necessary for normal growth and development of the central and peripheral nervous system^1^. In view of this, diseases of the thyroid gland can affect the functions of other organs. Thyroid diseases usually present clinically with features of hyperthyroidism, hypothyroidism, or as mass lesions^1^. They range from congenital lesions, hyperplastic, inflammatory, to neoplastic lesions. Surgical excision and histopathological evaluation are vital to establish a definitive diagnosis^1^,^2^.

The incidence of thyroid gland diseases varies with geographical location^3^,^4^. In Africa, dietary iodine deficiency is the major determinant of thyroid pathology, resulting in a spectrum of disorders^2^,^4^. Sex and age are also significant determinants. Thyroid disorders are more common in women and older individuals^4^,^5^.

Among the most prevalent non-neoplastic thyroid conditions is goitre, which refers to an abnormal enlargement of the thyroid gland^1^. This can be classified into diffuse goitre, involving uniform glandular enlargement, and nodular goitre, marked by focal hyperplastic nodules^1^. These hyperplastic disorders are often the result of iodine deficiency or dyshormonogenesis^1^. Goitre is extremely common throughout the world and is thought to affect more than 200 million individuals^1^,^5^,^6^. It is also by far the most commonly observed thyroid disorder in Africa^5^,^6^,^7^. It appears that the pattern of thyroid disorders in Africa is evolving with increasing iodine sufficiency, despite incomplete biomedical information^2^,^7^,^8^,^9^.

Other thyroid disorders, particularly carcinoma, inflammatory, and cystic lesions, also contribute significantly to the spectrum of thyroid pathology. Thyroid carcinoma, the most common endocrine malignancy, includes various histological types, such as papillary, follicular, medullary, and anaplastic carcinoma^1^,^7^. Papillary and follicular carcinomas are well-differentiated and usually have a good prognosis^1^,^7^. Medullary carcinoma, derived from parafollicular C cells, has a distinct pathogenesis, while anaplastic carcinoma is undifferentiated and carries a poor prognosis^1^,^7^. Additionally, poorly differentiated carcinoma and other rare variants are recognized based on distinct morphologic and molecular features^7^.

Inflammatory thyroid disorders, such as Hashimoto's thyroiditis and Graves' disease, are associated with autoimmune mechanisms.^1^ Cystic lesions, specifically thyroglossal duct cysts, are are also encountered in clinical practice and can present as nodules or mass lesions^1^.

In view of this apparent evolution of thyroid disorders in Nigeria and Africa at large, the aim of the study is to establish baseline data on frequency and histopathological patterns of thyroid lesions and their relationship with age and sex as seen in the histopathology department of a teaching hospital over an 11-year period (2012–2022)

## Methods and materials

This was a retrospective descriptive study. The materials for this study consisted of the slides and paraffin-embedded blocks of all the thyroidectomy specimens received in the histopathology department of a teaching hospital over a 11-year period from January 2012 to December 2022. For each case, the laboratory request form and duplicate copy of the histological report were retrieved. Clinical information such as age, sex, and the histological type of thyroid disease were extracted. All histology slides, stained with routine hematoxylin and eosin, were retrieved and studied. Fresh sections were made from formalin-fixed, paraffin-embedded tissue blocks where slides were missing or broken. All slides were reviewed microscopically by consultant histopathologists.

Inclusion criteria encompassed all thyroidectomy specimens received during the study period, regardless of age, sex, or histological diagnosis. No case was excluded, as none met the exclusion criteria of incomplete clinical data or inability to process or review the specimen adequately.

The cases were grouped into developmental/congenital, goitre/hyperplastic lesions, inflammatory, and neoplastic lesions. The neoplastic lesions were classified using the World Health Organization (WHO) classification of thyroid tumors^7^.

To ensure consistency in reporting, inter-rater reliability was maintained by having two independent histopathologists review a randomly selected subset of cases. While inter-rater agreement was not quantified statistically, discrepancies were resolved through discussion and consensus. Intrapersonal consistency was also ensured by adherence to standard diagnostic criteria, with consultation among specialists when necessary.

The diagnosis of Graves' disease was based on a combination of clinical presentation and characteristic histopathological findings. Histologically, Graves' disease is characterized by diffuse follicular hyperplasia with tall columnar epithelium, scalloping of colloid, papillary infoldings into the follicular lumina, and lymphoid aggregates in the stroma^1^.

Data was entered and managed using Microsoft Excel and Statistical Package for the Social Sciences (SPSS) software (IBM SPSS for Windows, Version 23.0). The data handling process was performed following standard protocols for accuracy and consistency. All patient identifiers were removed during data entry to maintain confidentiality. The age variable was grouped into decades (e.g., 0-9, 10-19, etc.) to facilitate analysis of trends across age groups. Sex was categorized as male or female.

Descriptive statistics were used to summarize the clinical and histological characteristics of the cases. The mean and standard deviation for age were calculated. Chi-squared tests were used to assess relationships between categorical variables. A p-value less than 0.05 was considered statistically significant. The results obtained were presented in tables and charts.

Permission for the conduct of this study was obtained from the Ethical Review Committee of the hospital (Protocol number: LTH/OGB/EC/2023/442). The study was conducted in accordance with the Declaration of Helsinki. All specimen evaluations were performed confidentially, and patient data were securely stored on a password-protected computer to ensure privacy and protection of personal health information.

## Result

**Figure 1:**
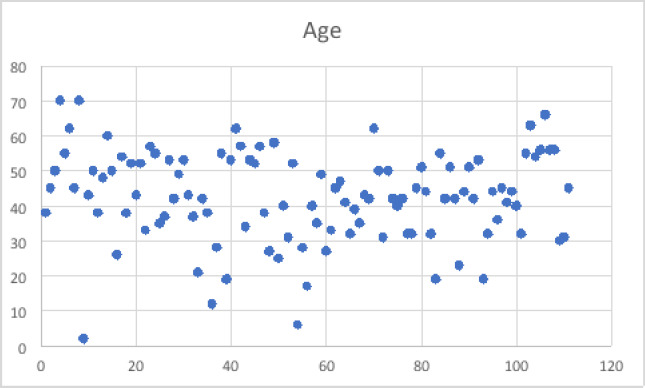
A scatter plot showing age of patients with thyroid lesions

**Figure 2:**
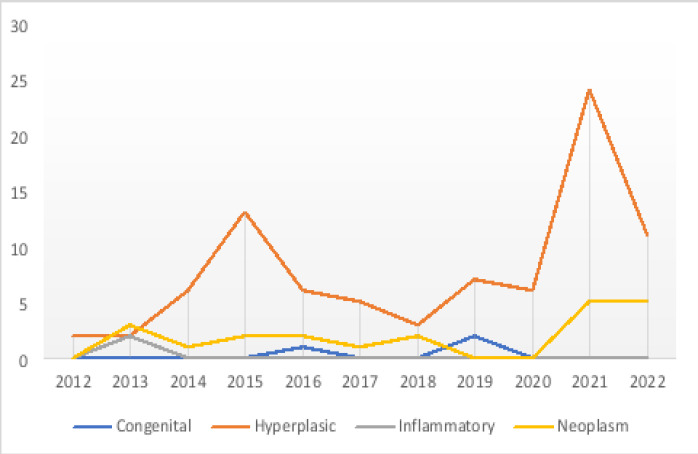
A graph showing yearly frequency distribution of thyroid lesions over the study period

A total of 111 thyroid specimens were received over the 11-year period, representing 2.4% of all the cases seen at the histopathology department. There were 94 females (86.7%) and 17 males (15.3%), giving a female to male ratio of 5.5:1 (Table 1). The overall age range was from 2 to 70 years, with a mean age at presentation of 42.5 years (SD = 12.94), and the peak age of incidence was seen in the 40-49 years age group (28.8%) (Figure 1 and Table 1). Trend analysis across all age groups showed an increased number of thyroid lesions between 2012 and 2015, followed by a dip between 2016 and 2018. In 2019, there was a spike, with a further dip in 2020 and 2022. Peak incidence was recorded in 2021 (Figure 2).

**Table 1:** Age group and aetiological classification of thyroid lesions

*Age group*	*Under 9*	*10 to 19*	*20 to 29*	*30 to 39*	*40 to 49*	*50 to 59*	*60 to 69*	*70 and above*	*Total*
*Congenital*	*1	2	0	0	0	0	0	0	3
	(0.9)	(1.8)	(0.0)	(0.0)	(0.0%)	(0.0)	(0.0)	(0.0)	(2.7)
*Hyperplastic*	0	0	5	19	28	28	5	0	85
	(0.0)	(0.0)	(4.5)	(17.1)	(25.2)	(25.2)	(4.5)	(0.0)	(76.6)
*Inflammatory*	1	0	0	0	1	0	0	0	2
	(0.9)	(0.0)	(0.0)	(0.0)	(0.9)	(0.0)	(0.0)	(0.0)	(1.8)
*Neoplasm*	0	3	3	6	3	3	1	2	21
	(0.0)	(2.7)	(2.7)	(5.4)	(2.7)	(2.7)	(0.9)	(1.8)	(18.9)
*Total*	2	5	8	25	32	31	6	2	111
	(3.6)	(4.5)	(7.2)	(22.5)	(28.8)	(27.9)	(5.4)	(1.8)	(100.0)

P-value=less than 0.001(confidence interval of 95%)

* Values in brackets indicate percentage

Hyperplastic lesions accounted for for 85 cases (76.6%), followed by neoplastic lesions, which accounted for 21 cases (18.9%). Congenital lesions and inflammatory lesions accounted for 3 (2.7%) and 2 (1.8%) cases, respectively. Hyperplastic lesions were predominantly observed in females (76.6%), with hyperplasia being most frequent in individuals aged 30 to 59 years. (Table 2) There was a statistically significant association between aetiological categories and both age and gender (p-value < 0.001) (Table 1, Table 2).

**Table 2:** Gender and aetiological classification of thyroid lesions

Aetiological	Female	Male	Total	Female: Male
Congenital	0(0.0%)	3(2.7%)	3(2.7%)	0:3
Hyperplastic	76(68.5%)	9(8.1%)	85(76.6%)	8.4:1
Inflammatory	1(0.9%)	1(0.9%)	2(1.8%)	1:1
Neoplasm	17(15.3%)	4(3.6%)	21(18.9%)	4.3:1
Total	94(84.7%)	17(15.3%)	111(100.0%)	5.5:1

P-value=less than 0.001(confidence interval of 95%)

Nodular hyperplasia (nodular goitre) was the most common histopathologic type, constituting 45% of all cases studied, with a female to male ratio of 6.1:1. Diffuse goitre was the second most common, accounting for 31.5% of all cases, with a female to male ratio of 16.5:1 (Table 3). Diffuse goitre was most commonly observed in the fifth decade, followed by the sixth decade, with frequencies of 12 (10.8%) and 11 (9.9%), respectively. Nodular hyperplasia was most common in the sixth decade, followed by the fifth decade, with frequencies of 17 (15.3%) and 16 (14.4%), respectively (Table 4).

**Table 3:** Gender and histopathology types of thyroid lesions

Female	Male	Total	Female: Male	
Congenital				
Thyroglossal cyst	0(0.0%)	3(2.7%)	3(2.7%)	0;3
Hyperplastic				
Diffuse goitre	33(29.7%)	2(1.8%)	35(31.5%)	16.5:1
Nodular hyperplasia	43(38.7%)	7(6.3%)	50(45.0%)	6.1:1
Inflammatory/autoimmunity				
Chronic non-specific thyroiditis	0(0.0%)	1(0.9%)	1(0.9%)	0:1
Grave disease	1(0.9%)	0(0.0%)	1(0.9%)	1:0
Neoplasm				
Follicular adenoma	1(0.9%)	1(0.9%)	2(1.8%)	1:1
Hyalinizing trabecular adenoma	0(0.0%)	1(0.9%)	1(0.9%)	0:1
Atypical follicular adenoma	0(0.0%)	1(0.9%)	1(0.9%)	0:1
Follicular tumor of uncertain malignant potential	1(0.9%)	0(0.0%)	1(0.9%)	1:0
Anaplastic carcinoma	1(0.9%)	0(0.0%)	1(0.9%)	1:0
Follicular carcinoma	4(3.6%)	1(0.9%)	5(4.5%)	4:1
Poorly differentiated carcinoma	2(1.8%)	0(0.0%)	2(1.8%)	2;0
Papillary thyroid carcinoma	8(7.2%)	0(0.0%)	8(7.2%)	8:0
Total	94(84.7%)	17(15.3%)	111(100.0%)	

P-value=less than 0.001(confidence interval of 95%)

**Table 4:** Age and histopathology types of thyroid lesions

	0 to 9	10 to 19	20 to 29	30 to 39	40 to 49	50 to 59	60 to 69	70 & above	Total
Congenital									
Thyroglossal cyst	*1	2	0	0	0	0	0	0	3
	(33.3)	(66.7)	(0.0)	(0.0)	(0.0)	(0.0)	(0.0)	(0.-)	(100.0)
Hyprplastic									
Diffuse goitre	0	0	2	9	12	11	1	0	35
	(0.0)	(0.0)	(5.7)	(25.7)	(34.3)	(31.4)	(2.9)	(0.0)	(100.0)
Nodular hyperplasia	0	0	3	10	16	17	4	0	50
	(0.0)	(0.0)	(6.0)	(20.0)	(32.0)	(34.0)	(8.0)	(0.0)	(100.0)
Inflammatory/autoimmunity									
CNST	1	0	0	0	0	0	0	0	1
	(100.0)	(0.0)	(0.0)	(0.0)	(0.0)	(0.0)	(0.0)	(0.0)	(100.0)
Grave disease	0	0	0	0	1	0	0	0	1
	(0.0)	(0.0)	(0.0)	(0.0)	(100.0)	(0.0)	(0.0)	(0.0)	(100.00
Neoplasm									
Follicular adenoma	0	0	0	1	0	1	0	0	2
	(0.0)	(0.0)	(0.0)	(50.0)	(0.0)	(50.0)	(0.0)	(0.0)	(100.0)
HTA	0	0	0	1	0	0	0	0	1
	(0.0)	(0.0)	(0.0)	(100.0)	(0.0)	(0.0)	(0.0)	(0.0)	(100.0)
AFA	0	0	1	0	0	0	0	0	1
	(0.0)	(0.0)	(100.0)	(0.0)	(0.0)	(0.0)	(0.0)	(0.0)	(100.0)
FTUMP	0	0	0	1	0	0	0	0	1
	(0.0)	(0.0)	(0.0)	(100.0)	(0.0)	(0.0)	(0.0)	(0.0)	(100.0)
Anaplastic carcinoma	0	0	0	0	0	0	1	0	1
	(0.0)	(0.0)	(0.0)	(0.0)	(0.0)	(0.0)	(100.0)	(0.0	(100.0)
Follicular carcinoma	0	0	0	1	1	2	0	1	5
	(0.0)	(0.0)	(0.0)	(20.0)	(20.0)	(40.0		(20.0)	(100.0)
PDC	0	0	1	0	0	0	0	1	2
	(0.0)	(0.0)	(50.0)	(0.0)	(0.0)	(0.0)	(0.0)	(50.0)	(100.0)
PTC	0	3	1	2	2	0	0	0	8
	(0.0)	(37.5)	(12.5)	(25.0)	(25.0)	(0.0)	(0.0)	(0.0)	(100.0)
Total	2	5	8	25	32	31	6	2	111
	(3.6)	(4.5)	(7.2)	(22.5)	(28.8)	(27.9)	(5.4)	(1.8)	(100.0)

P-value=less than 0.001(confidence interval of 95%)

* Values in brackets indicate percentage

CNST; Chronic non-specific thyroiditis, HTA; Hyalinizing trabecular adenoma, AFA; Atypical follicular adenoma, FTUMP; Follicular tumor of uncertain malignant potential, PDC; Poorly differentiated carcinoma, PTC; Papillary thyroid carcinoma.

Inflammatory thyroid lesions seen were Graves' disease and chronic non-specific thyroiditis. One case of Graves' disease was seen in a female patient in her fifth decade (Table 3, Table 4).

Thyroglossal duct cysts were the only congenital thyroid anomaly observed, accounting for 3 cases (2.7%). These were exclusively seen in males in the first two decades of life (Table 3, Table 4).

A majority of neoplastic cases (16/21) were carcinomas, with papillary thyroid carcinoma (PTC) being the most common (8 cases), followed by follicular carcinoma (5 cases). Two cases of poorly differentiated carcinoma and one case of anaplastic carcinoma were also noted. The peak incidence of PTC occurred in the second and fourth decades of life (Table 3, Table 4). There was a statistically significant association between histopathological types and age as well as gender (p-value < 0.001) (Table 3, Table 4)

## Discussion

Thyroid lesions are common worldwide, with varying prevalence and extent attributed to iodine deficiency and other environmental influences^1^. A total of 111 thyroid specimens were reviewed in this study, representing 2.4% of all the histopathological cases. This finding was higher than what was obtained in Zaria, Kano, and Port Harcourt, where thyroid lesions accounted for 1.1%, 1.5%, and 1.6% of histopathology cases seen in the department^2^,^3^,^8^. The higher prevalence in this study may be attributed to factors such as the iodine deficiency status in the region, or a higher rate of presentation to the tertiary hospital.

The overall age range was from 2 to 70 years. This finding was consistent with what was obtained in Port Harcourt^3^. Also, the finding of a mean age at presentation of 42.5 years (SD = 12.94) and peak age of incidence seen in the 40-49 years age group (28.8%) were similar to what was reported in a study in Yemen, where the mean age was 40.06 ± 13.18 years and peak incidence was in the fifth decade^11^. In contrast, the mean age of patients with thyroid diseases in Kano was 36.3 years, with the peak incidence (33.3%) seen in the age group of 30-39 years^2^. Our observations were also different from what was obtained in Lagos and Port-Harcourt^3^,^10^. These differences could be due to variations in sample sizes, healthcare access, and environmental influences, such as dietary patterns, or regional differences in iodine deficiency.

There were 94 females (86.7%) and 17 males (15.3%), giving a female-to-male ratio of 5.5:1, which compares favourably with 6.4:1, 7:1, 6.2:1, 6:1, and 5.7:1 from Kano, Lagos, Enugu, Ile-Ife, and Ilorin, respectively^2^,^10^,^12^,^13^. Similarly, studies from Ethiopia, Saudi Arabia, and Pakistan reported a female-to-male ratio of 4.5:1, 6.2:1, and 4.5:1, respectively^14^,^15^. Despite minor variation across studies, the consistent female predominance supports the role of hormonal and autoimmune factors in thyroid pathology.

In the present study, non-neoplastic lesions were found to be more common than neoplastic lesions, constituting 90 (81.1%) and 21 (18.9%) cases, respectively. A similar observation was noted in Nepal^16^.

Furthermore, hyperplastic lesions comprising both diffuse and nodular goitre accounted for 85 (76.6%) of all thyroid lesions. This finding was higher than what was reported from Zaria (72.1%), Ile-Ife (75%), Port Harcourt (59.4%), and Enugu (63.2%)^3^,^8^,^12^,^13^. In Ethiopia, Abebe and Osman reported that goitre accounted for 80% of all thyroid lesions^17^. These findings may be attributable to geographical location, socio-environmental factors such as dietary habits, and regional differences in iodine supplementation or deficiency.

In this review, the peak age of occurrence for goitre was in the fifth decade with a male-to-female ratio of 1:8.4. Raheem et al., Nggada et al., Nzegwu et al., and Ijomone et al. in their respective studies from Zaria, Ile-Ife, Enugu, and Port Harcourt, Nigeria, reported female preponderance of 1:9.2, 1:6, 1:6.4, and 1:10, respectively^3^,^8^,^12^,^13^. Also, Tsegaye and Ergete reported a male-to-female ratio of 1:4.5 in Ethiopia^5^.

Inflammattory lesions in the present study included Grave's disease and lymphocytic thyroiditis, one case each (0.9%), which accounted for only two cases of inflammatory or autoimmune diseases seen. The proportion of Graves' disease seen in this review was lower than findings from Zaria (4%), Enugu (3.1%), and Kano (5.4%), but closer to what was reported in 1.5% from Port Harcourt (1.5%), 1.4% from Ile-Ife (1.4%), and 0.91% from Karachi (0.91%)^2^,^3^,^8^,^12^,^13^,^15^ Compared to our study, lymphocytic thyroiditis was observed up to 1% and 15% in some studies^9^,^18^,^19^. The low detection rate may reflect underdiagnosis, as most autoimmune thyroid diseases are medically managed and may not require surgery.

Thyroglossal duct cysts were the most common congenital thyroid lesion seen, accounting for 2.7% of cases. They were seen exclusively in males in the first two decades of life. In Zaria, they accounted for 5.7% of the total cases studied, with the peak age of occurrence in the first decade of life and a male-to-female ratio of 1:1.1 in a review^8^. A study by Ijomone et al. showed a frequency of 5.3%, which was higher than what we observed in our finding but with a higher male-to-female ratio of 1:63. Our finding was higher than what was reported by Nzegwu et al. (1.8%) with a female-to-male ratio of 1:2 in a study^12^. Although congenital thyroid lesions are generally said to have neither sex predilection nor hereditary predisposition, our findings reveal otherwise^1^. This discrepancy may be due to sample size, as well as genetic and environmental factors that were beyond the scope of this study.

Thyroid gland neoplasms accounted for 18.9% and were the second most common thyroid diseases seen in this study. This finding was comparable to what was reported by Raheem et al. (16.8%) and Olatoke et al. (16.9%)^8^,^20^. However, Tsegaye and Ergete in Ethiopia and Ijomone et al. in Nigeria reported higher rates of 21% and 31.6%, respectively^3^,^5^. Our study showed a female preponderance of 4.3:1, as seen in the majority of similar studies on thyroid gland by Olatoke et al. from Ilorin and Ijomone et al. in Port Harcourt^3^,^5^,^20^. This female predominance was also seen in studies done in Pakistan (1:3.6) and Ethiopia (1:2.9)^5^,^15^.

Among the benign neoplastic lesions, we recorded 4 cases of follicular adenoma and a case of follicular neoplasm of undetermined malignant potential. The female-to-male ratio of 3:1 was comparable to 6:1 and 5.2:1 recorded in Port Harcourt and Zaria, respectively^3^. The reason could be due to our limited sample size.

Malignant lesions were seen in 14.4% of all the cases reviewed. All were carcinomas. This was similar to what was observed by Raheem et al. (12.6%), Nzegwu et al. (14.1%), and Ijomone et al. (15.1%)^3^,^8^,^12^. Papillary carcinoma was the most common carcinoma in this study, representing 53% of all malignant cases. Our finding was comparable to findings from Enugu (44.9%), Zaria (71.4%), and Port-Harcourt (55%)^3^,^8^,^12^. Peak incidences of papillary carcinoma were in the second and fourth decades with female preponderance. This observation was similar to what was obtained in Port-Harcourt and also agreed with other studies that concluded that papillary carcinoma commonly affects children and young adults^3^,^9^,^13^.

Follicular carcinoma accounted for 31.3% of the carcinomas with a female-to-male ratio of 4:1, with peak incidence in the 6th decade. Ijomone et al. in Port Harcourt also reported a female-to-male ratio of 5:1 and peak incidence in the 7th decade of life^3^. Globally and nationally, as indicated in the literature reviewed, papillary carcinoma was the most common histological variant, followed by follicular carcinoma^22^.

This study recorded only one case of anaplastic carcinoma (0.9%), which was similar to the reports of Nzegwu et al., Ngadda et al., and Raheem et al.^8^,^12^,^13^. In Yemen and the United States, anaplastic carcinoma accounted for approximately 2.85% and 1% of all thyroid malignancies, respectively^11^,^23^.

## Conclusion

This study confirms a clear female predominance in thyroid lesions and highlights the dominance of goitres, particularly in the fourth to sixth decades of life. Papillary carcinoma was the most frequent malignancy, seen predominantly in younger women. These findings are important for local clinical practice.

They underscore the need for targeted screening and preventive strategies, especially for middle-aged women, who are at higher risk of goitres and thyroid malignancy. Public health campaigns should promote early evaluation of thyroid enlargement and advocate for continued iodine supplementation. Future multicenter prospective studies with larger samples are recommended to better characterize trends and guide regional policy.

## Strengths and limitations

The limitation of the study was that it was carried out in a single tertiary hospital in South Western Nigeria. The findings may therefore not represent the entire population of the zone or the country. Another limitation is the retrospective nature of the study, which may have led to biased data. Furthermore, the sample size was relatively small, which could limit the generalizability of the results. There is also a potential selection bias, as only surgically treated lesions sent for histopathology were included. Despite these limitations, this study has provided baseline data for the histopathological patterns of thyroid lesions in our institution, and this shall be a basis for further work on thyroid lesions.

## Importance of Research Findings

This study is significant in that it provides an understanding of the patterns of thyroid lesions in a Nigerian population, which can aid in the development of targeted public health policies, including screening for thyroid diseases. The findings will also contribute to improving clinical practice by guiding the management of thyroid disorders, particularly in resource-limited settings. Furthermore, the research findings provide a valuable reference for other studies in this area, as they can help guide future investigations into the molecular and genetic factors influencing thyroid disease in Nigeria and similar regions.
